# The Massage Treatment in Dental Pathological Conditions

**Published:** 1890-10

**Authors:** W. F. Rehfuss


### International Dental Journal.

VOL. XI. OCTOBER, 1890. No. 10.

### Original Communications.[1]

### THE MASSAGE TREATMENT IN DENTAL PATHOLOGICAL CONDITIONS.[2]

BY W. F. REHFUSS, D.D.S.

THE therapeutic value of the massage treatment in various dental pathological conditions has not been recognized nor appreciated.

That it does possess important and reliable therapeutic qualities, which eventually must recommend its adoption, is not to be disputed. Its adaptability and success in dentistry must necessarily depend on the skill, tact, and judgment exercised by the operator in its application.

The area of our operations is limited to the mouth,—relatively a small field for the practice of massage in comparison with other portions of the body, as on the leg or arm, where massage is performed with ease.

When we consider the fact of the mucous membrane of the mouth being more delicate than the epidermis of the arm or leg, in conjunction with the small area for manipulation, the dentist is

[1] The editor and publishers are not responsible for the views of authors of papers published in this department, nor for any claim to novelty, or otherwise, that may be made by them. No papers will be received for this department that have appeared in any other journal published in the country.

[2] Read at a meeting of the Odontological Society of Pennsylvania, June, 1890.

confronted with a combination of difficulties that will require of him a greater degree of skill and care in manipulating than ordinarily, to derive the desired therapeutic benefit of the treatment, which, like all operations or treatments, can be learned and appreciated only by experience and practice.

Massage is derived from the Greek, *masso*, I knead, or handle; Arabic, *mass*, to press softly. It is a term generally accepted to signify a series of procedures which are best accomplished with the hands, such as friction, kneading, manipulating, rolling, and percussion of the external tissues of the body in a variety of ways, with either a curative, palliative, or hygienic object in view. In some crude form or other, massage has been practised from time immemorial by savage and civilized people. Those who have recorded their knowledge and appreciation of the value of massage have in almost every instance been men of eminence and renown, either as physicians, philosophers, poets, or historians, from the days of Hippocrates and Homer, who mentions it in the "Odyssey," eight hundred or one thousand years B.C., down to those of S. Weir Mitchell and Billroth in modern times.

Herodotus, the father of history, and others in the fifth century B.C. mention the advantages of external treatment of the human body, and, about a century later, the first rational information and treatise on this subject appeared in the aphorisms of Hippocrates.

And so on, through the ages, we find the subject mentioned in history and its use advocated by renowned men. Asclepiades, a celebrated Greek physician, used it in the years 128–156 B.C., and his popularity with the Romans was due to his success with it. It is a fact of history that Cicero, Julius Cæsar, and Martialis, of literary fame, were all warm advocates of its virtues.

The Greeks, Romans, Indians, Chinese, Japanese, and, in fact, the people of all nations of the world, have used massage in some form.

To Peter Henrik Link, of Sweden, credit is given for having instituted what are now well known as the Swedish movement cures.

In 1813 the Royal Central Institution was established at Stockholm, with the patronage of the Swedish government, in order that Link might practise and teach his system of massage and gymnastics. Critics endeavored to prove that his methods were but a revival of that of the Brahmins of India, of the Egyptian priests, of Asclepiades, of Hippocrates, Cleus, and other noted ancient physicians, and that all of the movements that Link advocated were described in an ancient book of the Chinese, the "Cong-Fou of the Tao′-Sse′." However, the merits of his system were proven by the establishment of institutions, similar to that at Stockholm, in other parts of Sweden and in other countries.

Many modern physicians of renown have, by their researches and zeal in advocating its therapeutic value, given the subject such a stimulus that its field of usefulness has been recognized and gradually increased, until it has found its way into general and special branches of medicine with success, and often when other means have failed. It only now remains for the dentist to adopt it in dental pathological conditions to make its application universal in the treatment of all portions of the body.

The Swedish method of manual treatment consists of different movements, termed Swedish movements, the object of which is to exercise the natural functions of a muscle, set of muscles, articulation, or limb, with the beneficial object to overcome existing unnatural or pathological conditions, and they may be either passive or active.

Under the head of passive movements we find the massage treatment classed. In the treatment or mode of application of massage we must, in a measure, follow the scientific methods of application advised and adopted by leading authorities on the subject in their treatment or manipulation of other parts of the body.

My especial methods of massage are based on those practised by Dr. Mezger, of Amsterdam; these he divides into four different manipulations, viz.:

*First*, *Effleurage* (centripetal stroking). According to Kurre Ostrom, this manipulation, performed on the arm or leg, consists of centripetal stroking (towards the heart). The stroking is performed with the palm or radial portion of the hand, tips of the fingers, or thumb. The movement is similar to a carpenter's plane, a forward and backward motion.

*Secondly*, Frictions, which are strong circular manipulations given with the thumb or tips of the fingers.

*Thirdly*, *Pétrissage* (kneading). This manipulation is performed generally by the thumb or index-finger. In general massage, an operator picks up a special tissue, muscle, or tendon, and, placing one finger on each side of the part, proceeds in centripetal motion with a firm pressure. The palm of the hand may also be used.

*Fourthly, Tapotement* (percussion), which is divided into four kinds:

(*a*) *Clapping*, performed with the palms of the hands.

(*b*) *Hacking*, with the ulnar border of the hand.

(*c*) *Punctation*, with the tips of the fingers.

(*d*) *Beating*, with the clinched hand.

In a modified form these four manipulations are applicable to massage on the oral mucous membrane.

In illustrating the mode of performing dental massage I will take a typical case, and describe the mánipulations and *modus operandi* adopted in the treatment of pathological conditions of that class.

*First*, Our diagnosis must be accurate, for upon that depends the nature of the manipulations, their force and frequency, and the length of time during which they are employed.

We will consider a case of acute periostitis, associated with pulpitis, caused by deposition of tartar around the neck of the tooth, and also from long-standing caries.

The periostitis developed and was followed by an acute attack of pulpitis. The tooth was a superior left first molar. The position of the manipulator is an important matter. In performing manipulations on the left side, the manipulator stands on the right side of his patient and uses the thumb and fingers of the right hand for manipulating, using the left-hand for the application of a napkin when necessary; and in manipulating the right side he stands to the left of his patient, using the fingers of his left hand if he finds that standing on the right side gives him a cramped and unnatural position.

The position of the hand is also of consequence. The operator uses the fore- or middle-finger, or both, for manipulating, supporting the thumb on the adjacent teeth, as a pivotal or fixed point, and it serves as a support and guide in directing the finger in the manipulating. The forefinger of the other hand serves to hold up the corner of the lip, if necessary. The thumb is used to perform massage, while the four fingers of the same hand rest on or grasp the cheek, also as a guide and support.

The introductory massage consists of *effleurage*,—centripetal stroking around the affected part, and not *directly* over the seat of inflammation. When swelling and pain are present, the centripetal stroking is commenced at the central incisors on the healthy tissue, using the thumb, fore-, or middle-finger for stroking. Continue this gentle stroking and gradually work towards the inflamed membrane. The healthy tissue beyond and around the second molar and wisdom tooth should be similarly treated. Besides the soothing effect, which enables us to approach gradually near the seat of the *periostitis*, and avoid causing much pain, the circulation is pushed along more rapidly, so that exudations are carried off more easily. It is a mistake to commence massage immediately upon an inflamed tissue. If we were to consult the patient's feelings, they would undoubtedly indicate to us to commence at a comfortable distance from the seat of inflammation, and, as has been stated, approach it gradually as the pain lessens and the swelling abates through the open absorbents. This removes the pressure from the terminal nerve filaments, and pain is relieved.

The experiments of Glax and Klemensiewiez have proved that in an inflamed region the lymph spaces are so choked up by the exudations and products of inflammation poured into them, in consequence of the abnormal activity of the vascular walls, that their outlets are insufficient; the efferent vessels are thus compressed and the circulation impeded, so that when we exert pressure upon this inflamed region, already suffering under partial compression of its efferent vessels, it may injure more than benefit. We can now use gentle stroking directly over the inflamed membrane, and follow this by deep manipulation, proceeding in the same manner as before,—gradually from the healthy tissue towards the seat of inflammation.

The greatest pressure should be towards the peripheral end and anterior and posterior to the inflamed membrane, and by thus gradually approaching it there is produced an anæsthetic effect, which lessens the pain; and comparatively firm pressure on the part can now be produced, which but a short time previous was very sensitive to the touch. The object of the *effleurage* (stroking) is to increase the circulation in the blood-vessels and lymphatics. When this stage of the operation has been reached, recourse to "*friction*" can be had, stroking in circles, starting from the anterior part of the mouth and manipulating towards the posterior portion, using the forefinger. This causes a loosening of the tissues, and the effusions and exudations to be spread over a greater surface and brought more easily into contact with the veins and lymphatics, and these are aided in their resorptive functions by the pressure and friction of massage.

After applying friction we again have recourse to centripetal stroking, which is always used before and after friction.

We now come to the third stage in manipulating,—applying *pétrissage* (kneading). This manipulation consists of a series of small circular movements performed by the forefinger or thumb, proceeding from the anterior to the posterior portion of the mouth; and it differs from friction, because in the latter very little pressure is exerted, friction being merely circular stroking manipulations, while *pétrissage* (kneading) consists of friction with an additional pressure. After performing *effleurage* and friction, I use a modified form of kneading, discarding the circular movements, as I find that by using plain centripetal stroking, and giving an intermittent pressure, kneading is more easily and efficiently accomplished. The gum now has a resisting surface—slightly roughened. By pressing the finger along the gum an intermittent jarring pressure is produced similar to that felt by the finger on a piece of rubber, on which, without pressing, a resisting and not a sliding motion is accomplished. It is unnecessary to use much pressure with this mode, as each resistance develops the succeeding pressure. *Tapotement* is the last manipulation. Its application was a difficulty not easily overcome, therefore I devised a small appliance for use in the dental hand-piece, and thereby accomplished the desired manipulation, which is a combination of clapping, hacking, and beating. The appliance consists of a mandrel or shaft of the same gauge as a bur, to fit in the hand-piece of the dental engine, at the other end is a small metal hub, and attached to the hub, at right-angle with the mandrel, are three pieces of elastic tubing, one-eighth inch in diameter and one inch in length, arranged like the spokes of a wheel, radiating from the centre. When this appliance is revolved in the hand-piece, the elasticity and flaccidity of the small tubes decrease the circumference, on account of the centripetal motion, and by holding it against the gum a light blow is given by each tube, the intensity of which is regulated by the speed of the engine. The object of this form of percussion is to reach the superficial vessels, and cause an influx of blood to the part. The aim of frictions and kneading is to squeeze the pathologically changed parts, and by carrying the diseased tissue into the healthy substances, expose them to a firm stroking, so as to have exudations absorbed by the lymphatics. It is a difficult matter to definitely state the length of time massage should be employed in manipulating the gum. I work from three to fifteen minutes, according to the object I wish to accomplish. It would require about eight minutes to perform the manipulations as just described.

The importance of extreme caution and gentleness in manipulating is not to be overrated, for therein lies the success of the treatment. By frequently questioning the patient we can learn whether the manipulating does or does not cause pain, and regulate the intensity accordingly, and thereby accomplish some relief and benefit; whereas, if the manipulator used undue strength upon such a sensitive tissue as the oral mucous membrane, he would injure rather than benefit the part by his treatment.

I will now consider the physiological effects of massage. Where there is an anæmic condition of the mucous membrane, the importance of some measure to overcome the inactivity and poor nourishment is not to be overestimated. The objective point is to attract the circulation to the parts where the anæmic condition exists. This is done by intermittent pressure of massage, which exerts a simultaneous influence upon all the tissues within its reach, the blood-vessels, lymphatics, nerves, etc. It causes a more rapid absorption of the products of waste, and stimulates and hastens the sluggish circulation. Gentle stroking, though soothing, is in a physiological sense a mild irritant to the superficial vessels. The explanation of the mechanical effect of centripetal stroking is to push along the circulation, and the collapsing vessels cause a suction that aids the returning current.

The outgoing or arterial circulation in the part thus treated is momentarily impeded, an accumulation of blood and a dilatation of the arterial walls taking place when pressure is used. On removing it, the increased volume of blood from accumulation rushes onward with greater force, owing to its sudden liberation into the partially emptied continuations of the arteries.

It is an accredited fact that in heat and massage we have two valuable remedial agents in the treatment of dental pathological conditions. More benefit can, no doubt, be derived from their use than is now accorded them. Moderate heat causes a fluxion of blood to the part, and dilates the vessels. It acts like cold if of a high temperature, causing a contraction of the vessels. To gain the desired therapeutic effect in the application of heat, the theory is to apply it of a moderate degree, 80° or 90° F., to the seat of the inflammation, and gradually increase the temperature.

The principle of massage is the same, for intermittent compression of massage causes a mechanical contraction and dilatation of all of the blood-vessels,—arterial, capillary, venous, and lymphatics,—at every application, from sixty times a minute and upward, and this is certainly sixty times oftener than could be caused by the variations of heat in the same time. The aid to the returning circulation by being pushed along by massage is much greater than that caused by heat, which has a tendency to enlarge the area of stasis, and the exudations are dispersed more rapidly than by heat. In the application of cold, pain and swelling can be reduced in inflammation, but instead of increasing the circulation in the part and hastening the returning current of blood as by massage, we do the reverse, the flow of blood is lessened, the veins and lymphatics are contracted, and the effused products are not easily absorbed, because of this contraction of the vessels, and of all the other tissues. Another danger of cold applications is that they may convert inflammation into gangrene, or may produce sloughing.

The dental pathological conditions in which massage is employed will now be considered, and at what stage it is beneficial. It must be remembered that, according to the requirements of each individual case, massage may be of primary or secondary importance, of no value at all, or even injurious. Briefly stated, it is beneficial and useful in local disturbances of the circulation and nutrition and of inflammations in their incipient stages or after the acute symptoms have passed away, as at the commencement of periostitis, gingivitis, at certain stages of acute or chronic *alveolar* abscesses, etc. Local congestion and irritation can be relieved by massage. In chronic alveolar abscesses the languid circulation can be aroused, which will aid the absorption of the products of waste. In the class of cases under consideration we have a sinus present, and a continual discharge of pus. It is assumed that the nerve-canals have been rendered thoroughly aseptic, and that no serumal or salivary deposits on the root or necrosis of the alveolus are present.

The condition described is a passive one, the tissue outside of the boundary of pus is torpid. If the granulations be allowed to remain in this condition, they may contract and consolidate in time, causing the sinus to close and the discharge of pus to cease; but we find from clinical experience that the tendency is to take on a retrograde action, gradually involving the surrounding tissue and alveolar process. We must take advantage of this passive stage, and produce absorption of some of these products of inflammation, and cause a consolidation or formation of new granulations. By massage an increased blood-supply is attracted to the parts, which removes the stasis, stimulates and produces activity generally; the tendency is to absorb the products of waste and the effusions. In conjunction with antiseptic treatment by injections, the prognosis is favorable. In acute alveolar abscesses, after the discharge has ceased, and the healing process commenced, massage is valuable, as the progress is more rapid than when massage is not used; but the manipulations should not be too vigorous; two or three treatments suffice in these cases.

Often a patient complains that in a tooth which has been abscessed but which is apparently cured, as the sinus has closed and the discharge of pus ceased, there is a sense of tenderness and soreness when the finger is pressed on the gum over the root of the tooth. This I have noticed is particularly the case in bicuspids and canines. There is a thickening and somewhat flabby condition of the tissues around the roots of the affected tooth. In several cases of this kind I have, by massage, in about three to six treatments, entirely removed such soreness to touch. The treatment of diseases of the peridental membrane, by this process, depends upon the severity of the inflammation. In the incipient stages the object is to abate the swelling, remove the stasis in the circulation, disseminate the products of inflammation, and to restore the normal circulation. The massage, though gentle at first, should be quite vigorous, as a counter-irritant effect is desired in the majority of cases. I have treated a number of cases of apical periostitis, due to the products of decomposition of the pulp penetrating the apical space, causing inflammation and the formation of gases. In these cases the usual treatment of opening the pulp-chamber, removing the products of decomposition, giving vent to the gas, and then inserting an antiseptic dressing in the canal was pursued, but the pain still persisted in a varying degree, but not as severe as before the treatment. Massage in each instance relieved pain and made the recovery more rapid than is usual in these cases. In various forms of periostitis, due to pulp inflammation, direct irritation of the pericementum, and other causes, I use only moderate massage. The objective point is to produce a slight counter-irritant effect, and to bring the blood into more active circulation, so that when medicines, such as tincture aconite, tincture iodine, chloroform, etc., are applied to the gum they are more easily absorbed. In fact, I always manipulate the gum before applying to it any remedies, as by massage an increased blood-supply is attracted to the parts, and consequently the amount of the medicine absorbed and carried to the seat of the inflammation is greater than ordinarily. In the treatment of periostitis by massage lancing is not necessary unless the local congestion is considerable; then it is advisable to use massage first, which increases the blood-supply and also removes stasis; therefore when lancing is performed the consequent flow of blood is greater and continues longer.

When the tendency of the inflammation is to the formation of pus, we can hasten it by vigorous application of the treatment, which softens the tissues and also, by reason of the heat created, tends to direct the pointing of the abscess at that part of the gum.

The treatment of dental neuralgia by massage depends upon the predisposing cause. If of local origin, due to the irritation of the periosteum of one or more teeth, climatic changes, etc., massage is usually efficacious, otherwise systemic remedies are indicated.

In anæmic conditions of the oral mucous membrane of persons who are of a neuralgic diathesis, massage is beneficial, because it increases and stimulates the local blood-supply. In the treatment of pericementitis and deep-seated pulpitis, much reliance is usually placed in the therapeutic value of counter-irritations. In this respect massage possesses virtues of great efficacy, because by it counter-irritation may be made as slight or vigorous as desirable. When we wish to produce strong counter-irritation, the manipulations are continued with more than usual vigor until a slight oozing of blood is produced by the rupture of the blood-vessels of the mucous membrane. In some persons the oral mucous membrane is more delicate, and therefore more prone to irritation than in others. When such is the case, before commencing the manipulations, the gum should first be rubbed with vaseline, lanoline, or albolene, but if the local irritation produced becomes annoying or painful, tincture krameria, acidi tannici and glycerin, or any astringent or demulcent wash will allay the inflammation. If care and gentleness are exercised in manipulating this will not be necessary.

In conclusion, I do not claim massage to be a specific remedy in the treatment of all dental pathological conditions, nor do I advise its use to the exclusion of all other remedies; but its application in the treatment within the past fourteen months of about one hundred and fifty cases, including pericementitis in various stages, acute and chronic alveolar abscesses, deep-seated pulpitis, various pathological conditions of the oral mucous membrane, etc., either alone or in combination with other remedies, and with more than average successful results, has proven to me that massage is and should be, if properly applied, a valuable adjunct to our many successful means of combating dental diseases.

